# Enhanced Effects of ISA 207 Adjuvant via Intradermal Route in Foot-and-Mouth Disease Vaccine for Pigs

**DOI:** 10.3390/vaccines12090963

**Published:** 2024-08-26

**Authors:** Ji-hyeon Hwang, Kwang-Nyeong Lee, Su-Mi Kim, Hyejin Kim, Sung-Han Park, Dong-Wan Kim, Giyoun Cho, Yoon-Hee Lee, Jong-Soo Lee, Jong-Hyeon Park

**Affiliations:** 1 Center for Foot-and-Mouth Disease Vaccine Research, Animal and Plant Quarantine Agency, 177 Hyeoksin 8, Gimcheon 39660, Republic of Korea; jihyeonh87@korea.kr (J.-h.H.); beliefsk@korea.kr (S.-M.K.); hyejin86@korea.kr (H.K.); shpark1124@korea.kr (S.-H.P.); dongwan12@korea.kr (D.-W.K.); libretto@korea.kr (G.C.); lyhee74@korea.kr (Y.-H.L.); 2 Veterinary College, Chungnam National University, Daejeon 34134, Republic of Korea; 3 Avian Influenza Research & Diagnostic Division, Animal and Plant Quarantine Agency, 177 Hyeoksin 8, Gimcheon 39660, Republic of Korea; leekwn@korea.kr

**Keywords:** adjuvant, foot-and-mouth disease, intradermal route, protection, vaccine

## Abstract

In South Korea, a mandatory nation-wide foot-and-mouth disease (FMD) vaccination policy is in place. However, a major side effect of the current method of intramuscular (IM) administration of oil-adjuvanted FMD vaccines is the formation of granulomas in the muscles of pigs. To address this issue, we assessed the possible application of intradermal (ID) vaccination. Initially, we compared the serological immune response in specific pathogen-free pigs inoculated with FMD vaccines formulated with eight different adjuvants, administered twice at the neck site using a syringe with a needle via the ID route. Among the formulations (water-in-oil-in-water (W/O/W), oil-in-water (O/W), and polymer nanomaterials), ISA 207 of W/O/W was the most effective in inducing immunogenicity followed by ISA 201 of W/O/W. ISA 207 was further tested in formulations of different antigen doses (12 or 1.2 μg) delivered via both IM and ID routes. All four treatments successfully protected the pigs against FMD virus challenges. To assess the feasibility of the field application of the vaccines with ISA 207, we conducted ID vaccination of conventional pigs using a needle-free device, resulting in the detection of significant levels of neutralizing antibodies. ISA 207 was shown to be superior to ISA 201 in inducing immunogenicity via the ID route. In conclusion, ISA 207 could be a suitable adjuvant for ID vaccination in terms of vaccine efficacy for FMD, allowing for alternate use of ID vaccination and subsequent reduction in the incidences of granuloma formation in the field.

## 1. Introduction

Foot-and-mouth disease (FMD) is caused by the foot-and-mouth virus (FMDV) and affects cloven-hoofed animals, including cattle, pigs, goats, and even wild animals such as yaks, bison, buffalo, and antelopes [1,2]. It is one of the most economically significant diseases of livestock worldwide [3]. FMD typically manifests with a fever and blisters on the mouth, tongue, snout, teats, and feet of cattle, sheep, and pigs [4]. The FMD virus belongs to the *Aphthovirus* genus of the *Picornaviridae* family and is classified into seven serotypes: O, A, Asia1, C, SAT1, SAT2, and SAT3. Notably, cross-protection between different serotypes is not possible, and depending on the difference of antigenicity, protection within the same serotype may be limited [5].

Effective control of FMD relies on vaccinating susceptible animals with inactivated FMDV [6,7]. Following a large-scale outbreak in 2010–2011, the Korean government has mandated the vaccination of all susceptible livestock against FMD since 2011. The vaccination policy entails repeated intramuscular (IM) injections of a vaccine containing an oil adjuvant, typically administered in the neck muscle using a syringe [8,9]. However, IM vaccination in pigs can lead to side effects, including local inflammation, swelling, and granuloma formation [10]. In South Korea, the price of the neck meat, the injection site of a vaccine, is notably higher than other parts of the pig. Therefore, the economic losses from the disposal of abnormal neck meats due to granulomatous tissues are estimated to be around USD 115 million per year [11].

Intradermal (ID) vaccination has emerged as an effective alternative to traditional needle-based IM vaccination methods. Numerous studies have explored the efficacy of ID vaccination against various diseases [12,13,14,15]. ID vaccination has the potential to reduce vaccine production costs by utilizing less antigen compared with IM vaccination while demonstrating comparable efficacy [16,17,18]. Additionally, ID vaccination of FMDV in pigs can mitigate the formation of granulomas at the injection site and streamline the vaccination process when employing needle-free injectors [17,19]. Previous studies have reported that FMD vaccination via the ID route in a needle-free delivery system elicited protective immunity against FMDV in pigs comparable to IM vaccination with the same amount of antigen [9,20]. Furthermore, it was confirmed that granuloma formation at the ID vaccination site was significantly reduced when compared with IM vaccination.

Herein, we employed a needle-based adjuvant screening method in pigs to identify an adjuvant suitable for ID vaccination and assessed the protective effect and immunogenicity of the vaccination by varying the antigen dose through the IM and ID routes. Based on the results, the efficacy of the ISA 207 adjuvant for ID vaccination was further elucidated using a needle-free injector in farm pigs.

## 2. Materials and Methods

### 2.1. Cell and Virus

Porcine kidney (LF-BK) cells were kindly provided by the Foreign Animal Disease Research Unit (FADRU), Plum Island Animal Disease Center (PIADC), Agricultural Research Service (ARS), United States Department of Agriculture (USDA), Greenport, New York, USA and utilized for virus isolation and neutralization tests. These cell lines were cultured in Dulbecco’s modified Eagle’s medium (DMEM; Thermo Fisher Scientific, Waltham, MA, USA) supplemented with 10% fetal bovine serum (Gibco, Waltham, MA, USA) and 1% Antibiotic-Antimycotic (Gibco, Waltham, MA, USA). The cells were maintained at 37 °C in a 5% CO_2_ incubator.

The A22 Iraq/24/64 (A22, GenBank accession No. AY593764.1) strain, acquired from the Pirbright Institute (WOAH/FAO reference laboratory for FMD, Woking, England, UK), was used for the virus neutralization test. Viral titers were determined and calculated using the Spearman–Karber method at a 50% tissue culture infective dose (TCID_50_) [21]. FMDV infection and culture were conducted at a Biosafety Level 3 facility of the Center for FMD Vaccine Research at the Animal and Plant Quarantine Agency (APQA).

### 2.2. Preparation of Experimental Vaccines

We obtained inactivated viral antigen of A22 Iraq/24/64 produced for the antigen bank by the Merial company (Pirbright, UK). Quantification of the viral antigen was conducted using a spectrophotometer (Ultrasepc^TM^ 8000, Little Chalfont, Buckinghamshire, UK)at an optical density of 259 nm for 146S virus particles. Images were captured under 100,000× magnification with transmission electron microscopy (TEM; Hitachi 7100, Tokyo, Japan).

A total of nine different vaccines were formulated with quantified 146S viral antigen and various adjuvant formulas according to the protocols provided by the manufacturers: 50% Montanide^TM^ ISA 206 VG (ISA 206; SEPPIC, Paris, France), 50% Montanide^TM^ ISA 201 VG (ISA 201; SEPPIC, Paris, France), 50% Montanide^TM^ ISA 207 VG (ISA 207; SEPPIC, Paris, France), 20% Emulsigen^®^-D (Emulsigen; MVP, USA), 20% Emulsigen^®^-D with 10% Rehydragel^®^HPA Aluminum hydroxide (Al(OH)_3_; General Chemical, Morris Plains, NJ, USA), 20% Rehydragel^®^HPA Aluminum hydroxide (Al(OH)_3_, General Chemical, NJ, USA), 20% Montanide^TM^ IMS1313 VG N (IMS 1313; SEPPIC, Paris, France), or 10% Montanide^TM^ GEL 01 PR (GEL 01; SEPPIC, Paris, France). The vaccines were formulated to contain an antigen dose of 12 or 1.2 μg/head.

### 2.3. Animal Experimentation

The animal experiments were conducted with the approval of the Institutional Animal Care and Use Committee of the APQA (IACUC approval number 2013-763, 2013-846, and 2015-203). For pigs, the vaccines were administered on the right side of the neck via ID or IM with different volumes per head (Table 1). For the ID route, a 19G needle or the Intradermal Application Liquids Injector (MSD, Rahway, NJ, USA) was utilized for the Mantoux method and needle-free method, respectively [22].

#### 2.3.1. Comparison of the Effects of Different Adjuvants for ID Vaccination in Specific Pathogen-Free Mini Pigs

For the first animal experiment (Exp 1), eighteen 8-week-old specific pathogen-free (SPF) pigs were purchased from OPTIFARM (Osong, Republic of Korea). Each pig was inoculated with one of the nine different experimental vaccines containing 12 μg of 146S antigen per dose (0.2 mL) via the ID route using a 19G syringe needle on the right side of the neck of two pigs per each group (Table 1).

At 8 weeks post-vaccination, all pigs received a booster dose of the vaccines using the same method as the primary vaccination. Serum samples were collected from the pigs at 0, 3, 4, 6, 8, and 12 weeks post-primary vaccination.

#### 2.3.2. Comparison of the Effect of Adjuvant ISA 207 in Different Delivery Conditions in SPF Mini Pigs

For the second animal experiment (Exp 2), fourteen 15-week-old SPF mini pigs were purchased from OPTIFARM (Osong, Republic of Korea). Twelve pigs were inoculated with one of the four different experimental vaccines formulated with ISA 207 via the ID or IM route on the right side of the neck. The remaining two pigs were inoculated only with the ISA 207 adjuvant without the viral antigen via the ID or IM route. Serum samples were collected at 0, 1, 2, and 3 weeks post-vaccination (wpv) by venipuncture (anterior vena cava) and placed into Vacutainer Serum Tubes (BD Biosciences, USA). Four weeks after vaccination, all pigs were bled and challenged with the A22 Iraq/24/64 virus at a dose of 10^5^ TCID_50_ in 100 μL, administered at two sites on the left hind foot pad. Nasal swabs and sera were collected from 0 to 10 days post-challenge (dpc) and additionally at 13 and 16 dpc. Nasal swabs were collected using the BD^TM^ Universal Viral Transport Kit (BD Biosciences, USA). Clinical signs were monitored for 16 days post-challenge, and the daily clinical symptoms in pigs were quantitatively determined by summing lesion or sign scores in each category according to Alves et al. [23].

#### 2.3.3. ID Vaccination in the Field Setting Using Selected Adjuvants and a Needle-Free Device with Pigs from an Ordinary Farm

For the third animal experiment (Exp 3), fifteen 8-week-old pigs were purchased from a commercial farm (Yesan, Republic of Korea). Ten pigs were inoculated with experimental vaccines containing 12 μg of 146S viral antigen per dose in 0.2 mL, formulated with ISA 207 or ISA 201, while the other five pigs were inoculated only with ISA 207 (Table 1). The inoculation route was ID, utilizing a commercial needle-free injection device, the Intradermal Application Liquids Injector (MSD, Rahway, NJ, USA), on the right side of the neck. Blood samples were collected, as in the preceding description.

### 2.4. Quantification of FMDV RNA in Serum and Nasal Swabs

Viral RNA was extracted from serum and nasal swab samples using an automated nucleic acid purification system (MagNA Pure 96; Roche Applied Science, Penzberg, Germany). Real-time reverse transcription polymerase chain reaction (rRT-PCR) was performed using the CFX96^TM^ real-time PCR system (Bio-Rad, Hercules, CA, USA). The rRT-PCR assay utilized the one-step prime-script RT-PCR kit (Bioneer, Daejeon, Republic of Korea) according to the manufacturer’s instructions. The sense and antisense primer sequences targeting the FMDV 3D region were 5′-GGAACYGGGTTTTAYAAACCTGTRAT-3′ and 5′-CCTCTCCTTTGCACGCCGTGGGA-3′, respectively. The probe sequence was 5′-CCCADCGCAGGTAAAGYGATCTGTA-3′; its 5′ end was labeled with 6-FAM and the 3′ end was labeled with TAMRA. Standard curves for quantification of viral genome copies in the samples were constructed using the Bio-RAD CFX Manager^TM^ version 3.1 program (Bio-RAD, Hercules, CA, USA). All reactions were performed in duplicates to ensure the accuracy and the reliability of results.

### 2.5. ELISA for Detecting FMDV Specific Sero-Conversion

The PrioCHECK FMDV SP A ELISA kit (Prionics AG, Schlieren-Zurich, Switzerland) was used to detect antibodies against structural proteins of FMDV in pigs. Serum samples were analyzed, and the final optical density (OD) values were expressed as percentage inhibition (PI) relative to the mean OD of the OD max control wells. Each ELISA test was conducted in duplicates.

### 2.6. Virus Neutralization Test

Neutralizing antibody titers were estimated using a microplate serum neutralization test (SNT) following the World Organization for Animal Health (WOAH) protocol (2022). Serum samples obtained from the whole blood of pigs were first inactivated at 56 °C for 30 min. Subsequently, the sera were serially diluted on a microplate. A viral suspension containing 100 TCID_50_ was then added to each well of the microtiter plate and incubated for 1 h. LF-BK cells were subsequently added to each well and incubated for 2–3 days to observe cytopathic effects resulting from the viral replication. The neutralizing antibody titers were calculated as the log10 of the reciprocal antibody dilution that neutralize the replication of the virus [24].

### 2.7. Statistical Analysis

Clinical scores, Ct values (or estimated numbers of viral genome copies in clinical samples), PI values, and SN titers were analyzed or visualized using GraphPad Prism version 8.4.3 (GraphPad Software, CA, USA). All data are presented as mean ± standard error of the mean (SEM). Statistical analyses were performed using ordinary one-way analysis of variance (ANOVA) followed by Tukey’s multiple comparisons test in the Prism software (version 9; GraphPad Software, San Diego, CA, USA). Significance levels were denoted as follows: * *p* < 0.05 (significant); ** *p* < 0.01 (very significant); *** *p* < 0.001 (highly significant); **** *p* < 0.0001 (extremely significant); and ns, *p* > 0.05 (not significant).

## 3. Results

### 3.1. Immunogenicity of ID Administration Using the FMDV Antigen with Eight Different Adjuvants (Exp 1)

**Figure 1:**
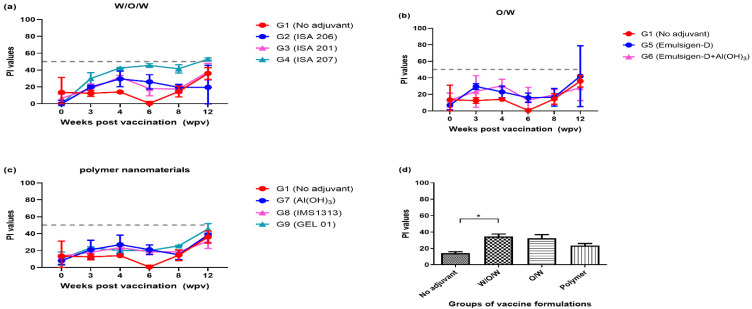
Comparison of SP antibodies (PI values) across nine groups using different adjuvant categories (Exp 1). (**a**) Comparison among the four groups, including the W/O/W formulation groups (G2, ISA 206; G3, ISA 201; G4, ISA 207) and G1 (no adjuvant). (**b**) Comparison among the three groups, including the O/W formulation groups (G5, Emulsigen-D; G6, Emulsigen-D with Al(OH)_3_) and G1 (no adjuvant). (**c**) Comparison among the three groups, including the polymer nanomaterials groups (G7, Al(OH)_3_; G8, IMS1313; G9, GEL 01) and G1 (no adjuvant). (**d**) Comparison of PI values at 8 wpv among three categories, including no adjuvant group. PI values ≥ 50% (dotted line) were considered positive for FMDV type A SP antibodies. SP; structure protein, PI; percentage inhibition, W/O/W, water-in-oil-in-water emulsion vaccines; O/W, oil-in-water emulsion vaccines; wpv, weeks post-vaccination; the datasheets are the mean ± SEM; statistical analyses were performed using one-way ANOVA; * *p* < 0.05.

**Figure 2:**
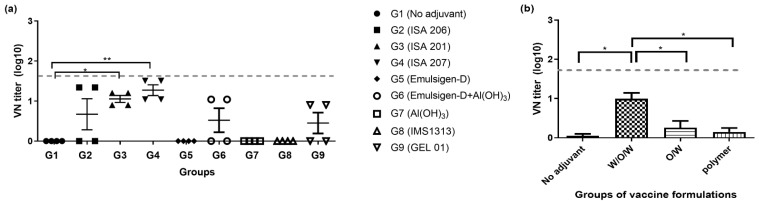
Neutralizing antibody titers of SPF mini pigs at 8 weeks post-vaccination (Exp 1). (**a**) VN titers against the A/Iraq/24/64 strain by each group: G1, (no adjuvant), G2 (ISA 206), G3 (ISA 201), G4 (ISA 207), G5 (Emulsigen-D), G6 (Emulsigen-D with Al(OH)_3_), G7 (Al(OH)_3_), G8 (IMS1313), G9 (Gel). (**b**) VN titers against A/Iraq/24/64 strain in each category. W/O/W adjuvant (G2, ISA 206; G3, ISA 201; G4, ISA 207), O/W adjuvant (G5, Emulsigen-D; G6, Emulsigen-D with Al(OH)_3_), polymer nanomaterials (G7, Al(OH)_3_; G8, IMS1313; G9, GEL 01). (**b**) VN titers against the A/Iraq/24/64 strain categorized by adjuvant types. VN titers ≥ 1.65 log_10_ (dotted line) are considered positive for FMDV antibodies. G, group; ID, intradermal; SPF, specific pathogen-free; W/O/W/, water-in-oil-in-water emulsion vaccines; O/W, oil-in-water emulsion vaccines; wpv, weeks post-vaccination; the datasheets are the mean ± SEM; statistical analyses were performed using one-way ANOVA; * *p* < 0.05; ** *p* < 0.01.

To evaluate the efficacy of adjuvants categorized by their formulation, the average PI values at 8 wpv of serum were analyzed across four categories: no adjuvant (G1, TN buffer), W/O/W formulation (G2, ISA 206; G3, ISA 201; G4, ISA 207), O/W formulation (G5, Emulsigen; G6, Emulsigen with aluminum hydroxide (Al(OH)_3_)), and polymer nanomaterials (G7, aluminum hydroxide (Al(OH)_3_); G8, IMS1313; G9, GEL 01). All groups showed a trend of decreasing PI values after 4 wpv. Specifically, in G4 (ISA 207), it was observed that the PI value approached 50 after the first vaccination and gradually decreased thereafter (Figure 1a–c). All groups showed increasing PI values after 8 wpv, which was estimated as the effects of boosted vaccines using the same method as the primary vaccination at 8 wpv. When comparing the PI values among treatment groups at 8 wpv, W/O/W formulations exhibited a similar trend to O/W formulations, but statistically significant differences were observed between W/O/W and the no adjuvant treatment group (Figure 1d). For the sera collected at 8 wpv, serum neutralizing antibody titers were measured against A22 Iraq/24/64, with G3 (ISA 201) and G4 (ISA 207) demonstrating the superior average VN titers of 1.05 (log10) or higher (Figure 2a). In the analysis of average VN titers across four categories of adjuvant groups, the W/O/W adjuvant group showed significantly higher values than all the other categories.

### 3.2. Clinical Score and Protection, in the Immunized and Challenged Pigs (Exp 2)

**Figure 3:**
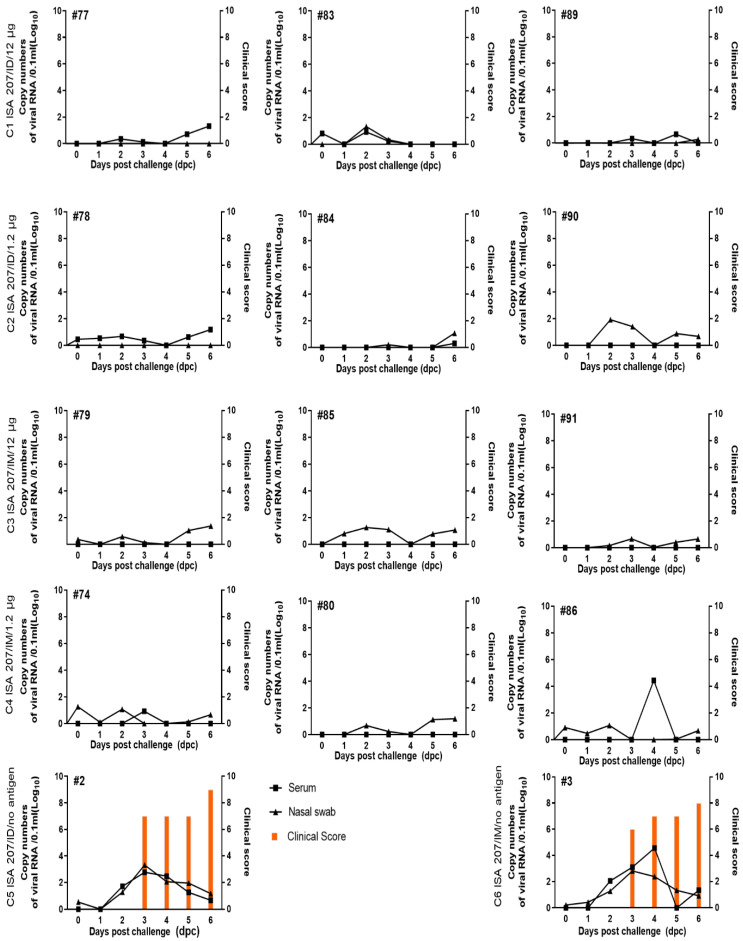
Virus excretion and clinical scores in SPF mini pigs immunized and challenged with the homologous virus (A/Iraq/24/64) (Exp 2). Nasal swabs and serum samples were collected from 0 to 10 and 13 and 16 dpc. Additionally, serum was collected at 0, 1, 2, and 3 wpv. The figure illustrates the virus excretion levels and clinical scores recorded throughout the experiment. C, challenge test group; ID, intradermal; IM, intramuscular; SPF, specific pathogen-free; dpc, days post-challenge; wpv, weeks post-vaccination.

Following the homologous challenge at 4 wpv in Exp 2, the four vaccinated groups (C1; ISA 207/ID/12 μg, C2; ISA 207/ID/1.2 μg, C3; ISA 207/IM/12 μg, C4; ISA 207/IM/1.2 μg) did not exhibit any clinical symptoms. Limited viral replication or shedding was observed in sera or nasal swabs in the four vaccinated groups, which were below 2 log10 copies of viral RNA in 0.1 mL of sample supernatant. Notably, pig #86 in C4 (ISA 207/IM/1.2 μg) showed transient viremia above 4 log10 viral RNA copies in 0.1 mL serum (Figure 3). In contrast, the pigs in C5 (ISA 207/ID/no antigen #2) and C6 (ISA 207/IM/no antigen #3), which were inoculated with the ISA 207 adjuvant without FMDV antigen, exhibited eight to nine clinical scores of FMD (fever and typical disease-related symptoms) within three days after the challenge (Figure 3 and Table S1).

**Figure 4:**
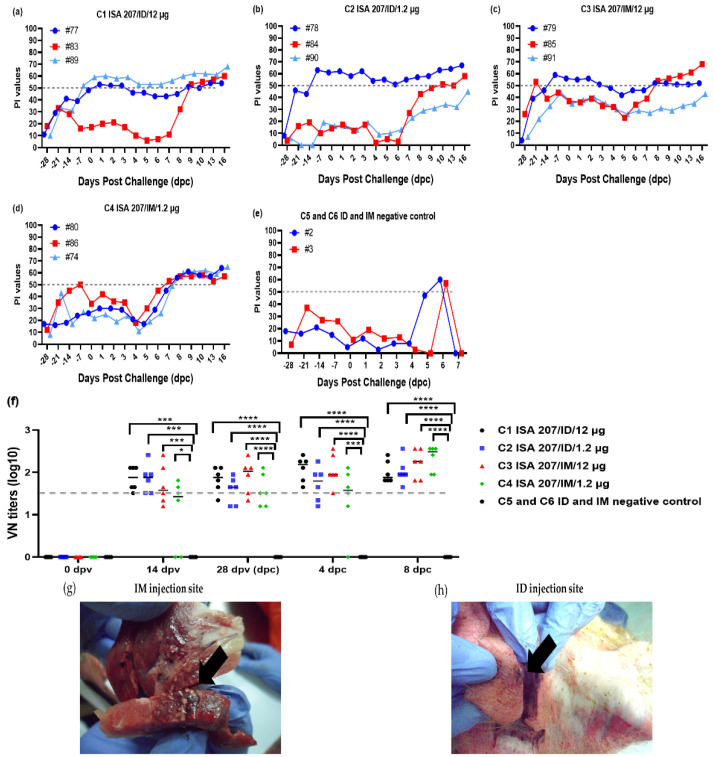
SP antibody (PI) and neutralizing antibody (VN titer) of SPF mini pigs in six challenge test groups vaccinated and bled at 0, 7, 14, 21, and 28 dpv and 0–16 dpc (Exp 2). (**a**–**e**) SP antibody responses in SPF mini pig sera collected at 0, 7, 14, 21, and 28 dpv and 0–16 days dpc. (**f**) VN titers (log10) of serotype A/Iraq/24/64 at 4 wpv. (**g**,**h**) Comparison of vaccine injection sites sacrificed at 16 dpc. The IM injection site exhibited nodular lesions in the neck muscle, whereas the ID injection site showed redness and small swelling on the skin. PI values ≥ 50% (dotted line) were considered positive for FMDV type A SP antibodies. VN titers ≥ 1.65 log_10_ (dotted line) were considered positive. C, challenge test group; ID, intradermal; IM, intramuscular; SPF, specific pathogen-free; SP, structure protein; PI, percentage inhibition; wpv, weeks post-vaccination; dpc, days post-challenge; the datasheets are the mean ± SEM; statistical analyses were performed using one-way ANOVA; * *p* < 0.05; *** *p* < 0.001; and **** *p* < 0.0001.

The pig sera collected after vaccination and viral challenge were tested with the SP ELISA, revealing a clear increase in weekly PI values in six pigs (C1; #77, #83, C2; #78, C3; #85, #79, #91, and C4; #80) compared to the control group vaccinated only with adjuvants (Figure 4a–d). A sharp increase in PI values was observed around 6–8 dpc in the vaccinated pigs (C1; #89, C2; #84, #90, and C4; #80, #86, #74), which had lower structural protein antibodies at 4 wpv (Figure 4a–d).

At 0 days post-challenge (4 wpv), the average neutralizing antibody titer in groups C1 to C4 were 1.4 log10 or higher, significantly exceeding the average titer of the C5 and C6 groups, which were negative control groups (**** *p* < 0.0001) (Figure 4f). The strength of antibody titers and the rate of seroconversion showed a dose-dependent pattern, demonstrating the superior average SP values and VN titers (C1; 41 P1 values and 1.83 log10 VN titers, C2; 30 P1 values and 1.58 log10 VN titers, C3; 43 P1 values and 1.90 log10 VN titers, C4; 27 P1 values and 1.60 log10 VN titers), regardless of adjuvant type used (Figure 4 and Table S1).

In the pigs vaccinated via the IM route by syringe (C3 and C4), granulomas were observed in the neck meat at 16 dpc (Figure 4g). In contrast, no granulomas were observed in the neck meat of pigs that were vaccinated with the same vaccines via the ID route (C1 and C2), likely due to the vaccines being delivered to the dermis or only to the skin layers (Figure 4h).

### 3.3. Serological Response in Domestic Pigs Vaccinated Using Selected Adjuvants with a Needle-Free ID Injector (Exp 3)

**Figure 5:**
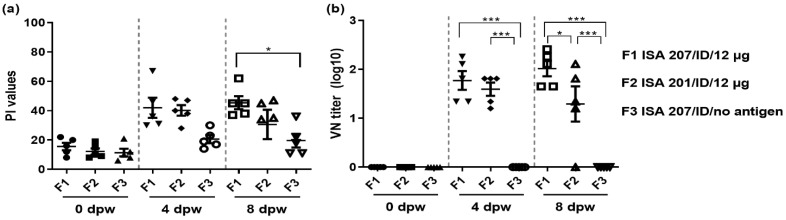
SP antibody response and neutralizing antibody response in domestic pigs’ sera at 0–8 wpv. The graphs illustrate the SP ELISA PI values and VN titers for each farm pig group (F1, ISA 207/ID/12 μg; F2, ISA 201/ID/12 μg; F3, ISA 207 adjuvant/ID/no antigen). (**a**) SP ELISA antibody level as measured by PI values ≥ 50% (dotted line) are considered positive for FMDV type A SP antibodies. (**b**) VN titers (log10) for the serotype A/Iraq/24/64 antibody values ≥ 1.65 log10 (dotted line) are considered positive for FMDV antibodies. F, farm pig group; ID, intradermal; SP, structure protein; PI, percentage inhibition; wpv, weeks post-vaccination; the datasheets are the mean ± SEM; statistical analyses were performed using one-way ANOVA; * *p* < 0.05; *** *p* < 0.001.

In Exp 3, all 15 pigs across three groups were bled at 0, 4, and 8 wpv and tested for SP antibodies and neutralizing antibodies (Figure 5). At 8 wpv, the average PI value of F1 (ISA 207/ID/12 μg, 45 PI value) was significantly higher than that of F2 (ISA 201/ID/12 μg, 31 PI value) and F3 (ISA 207/ID/no antigen, 19 PI value) (* *p* < 0.05) (Figure 5a). Similarly, the mean VN log10 values for sera collected at 8 wpv were 2.02 and 1.50 for F1 and F2, respectively, which were significantly higher than those of the control groups (*** *p* < 0.001). Furthermore, F1 showed slightly higher neutralizing antibody titers than F2 (* *p* < 0.05) (Figure 5b).

## 4. Discussion

Inactivated FMDV vaccines are typically formulated with adjuvants such as Al(OH)_3,_ saponin, or oil-based emulsions [25]. Gel vaccines, although effective in cattle and ruminants, often provide a shorter duration of immunity in pigs. Consequently, oil adjuvants have been used since the 1970s to enhance immune responses in pigs [26]. These oil-adjuvanted vaccines are known for inducing robust and long-lasting humoral immunity, which is crucial for extended protection in both pigs and cattle [25]. Different types of oil adjuvants are available, including W/O/W, W/O, and O/W types, each with distinct safety and tolerability profiles [26,27,28].

The immune activation induced by adjuvants in vaccines can sometimes lead to adverse side effects. For example, excessive reactions from IM injections can result in granulomas, sterile abscesses, and muscle nodules [29,30]. In South Korea, previous research on ID vaccination for FMDV in pigs has shown promising results [9].

In pigs, it has been reported that ID vaccination with only 1/10th of the normal IM dose can elicit a comparable level of protective efficacy. Similarly, in cattle, ID vaccination with only 1/16th of the normal IM dose provided protective immunity by 7dpv [16,18]. Experimental FMD vaccines with various adjuvant formulations for ID vaccination in pigs have been tested and compared [9,20]. These studies, which employed different types of needle-free devices and adjuvants, showed that ID vaccinations were as successful as IM vaccinations in protection against challenges. However, in terms of humoral immunity, ID vaccination resulted in similar or lower antibody levels compared to IM vaccination. Previous research has reported that ID vaccination is protective against challenges despite lower levels of humoral antibodies when compared to IM vaccination. This protection is attributed to an increased cell-mediated immune response, which induces the recruitment of inflammatory dendritic cells [17].

For the field application of ID vaccination, needle-free injectors could be considered due to several advantages over traditional needle-based ID vaccinations. These advantages include increased efficiency in terms of time and labor as well as enhanced safety of workers and consumers by eliminating the use of needles [31,32,33]. Previous studies on other diseases have investigated the feasibility of needle-free injectors for vaccination in pigs [34,35]. However, for FMD, despite interest in ID vaccination, studies on the formulation of optimal vaccines for ID vaccination in practical settings using needle-free injectors have been limited.

Thus, we evaluated the humoral immunogenicity of experimental vaccines composed of FMDV-inactivated serotype A antigen and various adjuvants, administered via the ID route to SPF mini pigs. Among the tested adjuvants, W/O/W-based adjuvants, ISA 201 and ISA 207, demonstrated superior performance for ID vaccines when compared with O/W and polymer nanomaterial-based adjuvants. The immune-stimulating effects in ID vaccination was most pronounced in groups using ISA 207 as an adjuvant.

Based on these results, SPF mini pigs were vaccinated with different quantities of antigen formulated with ISA 207 via ID or IM routes. All the vaccinated groups were clinically protected against FMDV, although there was an antigen dose dependency in the strength of the elicited immunity. We concluded that both routes, ID and IM, are effective for protection, with comparable antibody titers between them. Importantly it was demonstrated that ID vaccination could potentially avoid or reduce the formation of granulomas in the meat at the injection site.

In the experiment to determine suitable adjuvants for ID vaccination in the field, pigs from a commercial farm were vaccinated with inactivated antigen formulated with ISA 201 or ISA 207 using a new needle-free ID injector. ISA 207 was demonstrated to be the most suitable adjuvant candidate for the ID vaccination of FMD in pigs.

## 5. Conclusions

In our study, we found that the ISA 207 adjuvant was consistently the most effective adjuvant for ID vaccination in pigs for FMD. It conferred protective and neutralizing antibody titers comparable to those achieved with IM vaccination when the same amount of antigen was used. Further research is warranted to explore the potential for the field application of needle-free ID delivery of the FMD vaccines in pigs.

## Figures and Tables

**Table 1:** Overview of experimental vaccination protocols to enhance immune responses in pigs with various adjuvants via IM and ID routes.

Exp No.	Groups	Identification of Pigs	Antigen Payload (μg/head)	Adjuvants	Administration Route	Injected Volume/Dose (mL)
Exp1 ^SPF/N^	G1	#26, #20	12	No	ID	0.2
	G2	#19, #21	12	ISA 206	ID	0.2
	G3	#23, #24	12	ISA 201	ID	0.2
	G4	#25, #11	12	ISA 207	ID	0.2
	G5	#8, #12	12	Emulsigen-D	ID	0.2
	G6	#27, #22	12	Emulsigen-D + Al(OH)_3_	ID	0.2
	G7	#9, #15	12	Al(OH)_3_	ID	0.2
	G8	#17, #13	12	IMS1313	ID	0.2
	G9	#16, #14	12	GEL 01	ID	0.2
Exp2 ^SPF/N^	C1	#77, #83, #89	12	ISA 207	ID	0.2
	C2	#78, #84, #90	1.2	ISA 207	ID	0.2
	C3	#79, #85, #91	12	ISA 207	IM	1
	C4	#80, #86, #74	1.2	ISA 207	IM	1
	C5	#2	No	ISA 207	ID	0.2
	C6	#3	No	ISA 207	IM	1
Exp3 ^FP/NFD^	F1	#2, #3, #6, #9, #10	12	ISA 207	ID	0.2
	F2	#21, #22, #23, #27, #29	12	ISA 201	ID	0.2
	F3	#14, #15, #16, #18, #19	No	ISA 207	ID	0.2

Exp, experiment; SPF, specific pathogen-free; FP, farm pig; N, 19G needle; NFD, needle-free device; G, groups; Al (OH)_3_, aluminum hydroxide; ID, intradermal; IM, intramuscular.

## Data Availability

The original contributions presented in the study are included in the article/Supplementary Material, further inquiries can be directed to the corresponding authors.

## Supplementary Materials

The following supporting information can be downloaded at https://www.mdpi.com/article/10.3390/vaccines12090963/s1: Table S1: Summary of clinical signs and laboratory tests in vaccinated and challenged SPF mini pigs.
