# Supplementary material for: Enhanced Effects of ISA 207 Adjuvant via Intradermal Route in Foot-and-Mouth Disease Vaccine for Pigs
Source: Vaccines (Basel). 2024 Aug 26;12(9):963. doi: 10.3390/vaccines12090963 (PMC11435775; doi:10.3390/vaccines12090963)
Supplement: Supplementary file 1 [file vaccines-12-00963-s001.zip › vaccines-3077384-supplementary.pdf]

Table S1. Summary of clinical signs and laboratory tests in vaccinated and challenged SPF mini pigs

Vaccinated groups (adjuvant/rout/μg)	Pig ID	VN titer against A22 Iraq/24/64 at 28 dpv	Protective effects after challenge*		Virus detection by rRT-PCR for 6 days after challenge	
			Fever T > 40°C	Clinical score	Nasal swabs	Sera
C1(ISA 207/ID/12 μg)	#77	1.65±0.31	−	0	−	+
	#83	1.96±0.15	−	0	−	−
	#89	1.88±0.23	−	0	+	−
C2(ISA 207/ID/1.2 μg)	#78,	1.80±0.15	−	0	−	+
	#84	1.20±0.00	−	0	+	+
	#90	1.73±0.08	−	0	+	−
C3(ISA 207/IM/12 μg)	#79	2.03±0.08	−	0	+	−
	#85	1.42±0.08	−	0	+	−
	#91	2.26±0.15	−	0	+	−
C4(ISA 207/IM/1.2 μg)	#80	1.43±0.22	−	0	+	−
	#86	1.66±0.15	−	0	+	−
	#74	1.73±0.22	−	0	+	−
C5(ISA 207/ID/no antigen)	#2	<1.20±0.00	+	9	+	+
C6(ISA 207/IM/no antigen)	#3	<1.20±0.00	+	8	+	+

SPF, specific pathogen-free; C, challenge test group; ID, intradermal; IM, intramuscular; VN, virus neutralizing, rRT-PCR, real-time reverse transcription-polymerase chain reaction; dpv, days post-vaccination, dpc, days post-challenge; FMD, foot-and-mouth disease.

* challenged with A22 Iraq/24/64 at 28 dpv and examined until 16 dpc; † Clinical scores were based on the sum of each FMD lesion or sign (maximum score = 16) according to the method reported by Albes et al. based on the addition of point.
